# Injury Patterns and Demographics in Child and Adolescent Assault Victims Presenting to US Emergency Departments

**DOI:** 10.1155/2020/8169030

**Published:** 2020-10-24

**Authors:** Randall T. Loder, Samantha Palma, Maddie Smith

**Affiliations:** Department of Orthopaedic Surgery, Indiana University School of Medicine, Indianapolis, IN, USA

## Abstract

**Objective:**

To correlate injury patterns with patient demographics in child and adolescent assault victims.

**Methods:**

The National Electronic Injury Surveillance System-All Injury Program data for the years 2005 through 2015 was used. Injuries due to assault were identified and analyzed with SUDAAN 11.0.01™ software to account for the weighted, stratified nature of the data.

**Results:**

There were an estimated 4,407,009 ED visits for assault in patients ≤ 19 years of age. With increasing age, the percentage of females decreased. Sexual assaults were more common in females (87.4%), and robbery/burglary was more common in males (79.8%). When the perpetrator was a spouse/partner, the assault victim was most commonly female (88.8%), and when a stranger, the assault victim was most commonly male (71.5%). With increasing age, the percentage of sexual assaults decreased while the reason for the assault being unknown increased. The assault occurred in the home in 59.6% of those ≤ 4 years of age, decreasing to 18.7% in those 15 to 19 years of age. The anatomic location was the head/neck in 32.8% of those ≤ 4 years of age, increasing to 60.6% in those 15-19 years old. Those ≤ 4 years old had the highest hospital admission rate (8.3%). The main diagnoses were concussion (3.0%), contusion/abrasion (33.3%), fracture (11.5%), laceration (11.5%), internal organ injury (11.5%), puncture (2.8%), and strain/sprain (20.7%). The number of assaults from 2005 to 2015 decreased for all age groups except for those ≤ 4 years old.

**Conclusions:**

These data provide a comprehensive overview of child and adolescent assault victims presenting to the ED in the USA and can be used as background data for further study. The decreasing numbers of assaults over the 11 years of the study are encouraging, and challenges still exist in decreasing the number for those ≤ 4 years old.

## 1. Introduction

Violence and assault are significant public health issues [1]. Although they have been studied in the general population, there are few overall studies of assault in children and adolescents that correlate injury patterns with the demographics of age and gender. Most studies in children and adolescents only address particular anatomic areas (e.g., craniofacial) or singular mechanisms of injury (firearm injuries, sexual assault, and nonaccidental trauma). Barmparas et al. [2] studied children admitted to a trauma center for assault using the National Trauma Data Bank; however, such results are skewed to more serious injuries as these children were seen at a major trauma center. Mollen et al. [3] reviewed youth violence patients presenting to three hospitals in the Philadelphia area, but the study was limited by age (8 to 24 years), as well as urban location. The purpose of this study was to analyze all child/adolescent assault patients in the USA presenting to emergency departments (ED) and correlate the injury patterns with patient demographics by age and gender. Such information will be very useful to all health care providers involved in caring for injured children and adolescents. Understanding injury patterns with their associated demographics will be helpful information for such a health care provider by giving clues to the potential for assault when not immediately divulged by the patient.

## 2. Materials and Methods

Children and adolescents were defined as individuals ≤ 19 years of age. The National Electronic Injury Surveillance System- (NEISS-) All Injury Program (AIP) data was used for this study. The NEISS is a stratified, weighted dataset managed by the US Consumer Product Safety Commission (USCPSC) which collects injury data from ~100 hospitals in the United States and its territories having an emergency department (ED). It was initially designed for injuries due to consumer products. However, not all injuries are from consumer products; thus, the USCPSC selected ~65 of these hospitals (actual numbers vary slightly from year to year) to obtain data for all nonfatal injuries, regardless of the association with consumer products. This has been designated the All Injury Program (AIP). This data is in the public domain and housed by the Inter-University Consortium for Political and Social Research (ICPSR). It can be accessed at https://www.icpsr.umich.edu/icpsrweb/ICPSR/search/studies?q=all+injury+program. Use of this publicly available deidentified data was considered exempt by our local Institutional Review Board.

The database includes date of ED visit, gender/race/age of the injured patient, diagnosis, disposition from the ED, incident locale, body part injured, perpetrator and type of assault, reason for assault, causative agent/event of the injury, and hospital size (strata). Detailed descriptions of these variables are given in the appendix. The NEISS-AIP data for the years 2005 through 2015 was used. These years were chosen because 2015 was the last available year at the time the study began in mid-2019, and data before 2005 was coded differently for many variables, making it difficult to combine the years before 2005 with those afterwards. Injuries due to assault were identified using the code INTENT=1 (assault). Race was classified as White, Black, Amerindian (Hispanic and Native American), and Asian [4]. NEISS does not code for Polynesian and Indo-Mediterranean peoples.

### 2.1. Statistical Analysis

Statistical analyses were performed with SUDAAN 11.0.01™ software (RTI International, Research Triangle Park, North Carolina, 2013) which accounts for the weighted, stratified nature of the data. The estimated number of injuries/ED visits is calculated, along with 95% confidence intervals (CIs) of the estimate. When the actual number of patients (*n*) is <20, the estimated number (*N*) becomes unstable and should be interpreted with caution; thus, we report both the *n* and *N*. The annual incidence of ED visits for assault was calculated using the US Census Bureau data. Analyses between groups of continuous data were performed with the *t*-test (2 groups) or ANOVA (3 or more groups). Differences between groups of categorical data were analyzed by the *χ*^2^ test. A *p* < 0.05 was considered to be statistically significant.

## 3. Results

There were a total of 5,702,369 ED visits in the NEISS-AIP database from 2005 through 2015, for an estimated 337,627,315 patients. Of these estimated 337,627,315 patients, there were an estimated 18,116,132 (14,855,602–22,013,301) due to assault (5.4%). Of these assault patients, an estimated 4,407,009 (4,139,536–4,686,643) were in those ≤ 19 years of age (24.3% of all assault patients).

The average age of these 4,407,009 patients was 14.5 years. Age group distribution was 286,883 (228,316–359,410) (6.5%) (≤4 years), 322,164 (282,865–366,449) (7.3%) (5 to 9 years), 995,807 (922,941–1,072,512) (22.6%) (10 to 14 years), and 2,794,290 (92,693,157–2,892,438) (63.5%) (15 to 19 years old). There were 2,653,938 (60.2%) males and 1,752,659 females (39.8%). Racial composition was known in 3,506,513 patients: White in 1,517,881 (993,044–2089,531) (43.3%), Black in 1,264,225 (974,460–1,586,346) (36.1%), Amerindian in 692,581 (351,002–1,236,046) (19.8%), and Asian in 31,826 (16,831–59,961) (0.9%). Disposition from the ED was known in 4,329,619 patients: 4,110,966 (3,997,537–4,187,175) (94.9%) were treated and released and 281,653 (142,444–332,082) (5.1%) were admitted to the hospital.

### 3.1. Analyses by Gender

**Figure 1:**
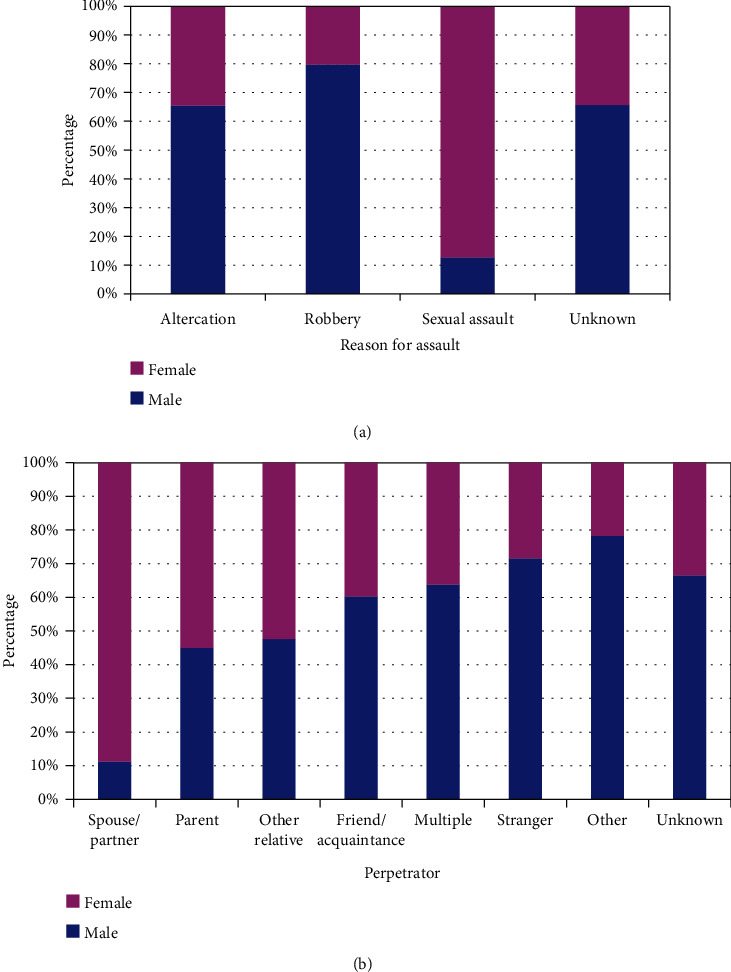
Differences by gender in child and adolescent assault victims. (a) By reason for assault (*p* < 10^−4^). (b) By perpetrator of assault (*p* < 10^−4^).

There were differences by gender for all demographic variables (Table 1). With increasing age, the percentage of female patients decreased. Sexual assaults occurred most commonly in females (87.4%), and robbery/burglary most commonly in males (79.8%) (Figure 1(a)) When the perpetrator was a spouse/partner, the assault victim was most commonly female (88.8%); when it was a stranger, the assault victim was most commonly male (71.5%) (Figure 1(b)). When the assault occurred at home, the victim was female in 55.9%, and when on the street, the victim was male in 71.0%. When the injury involved the lower trunk, 70.5% of the victims were female, and when the upper extremity, only 32.8% were female. When the victim was admitted to the hospital, 78.8% were male.

### 3.2. Analyses by Age Groups

**Figure 2:**
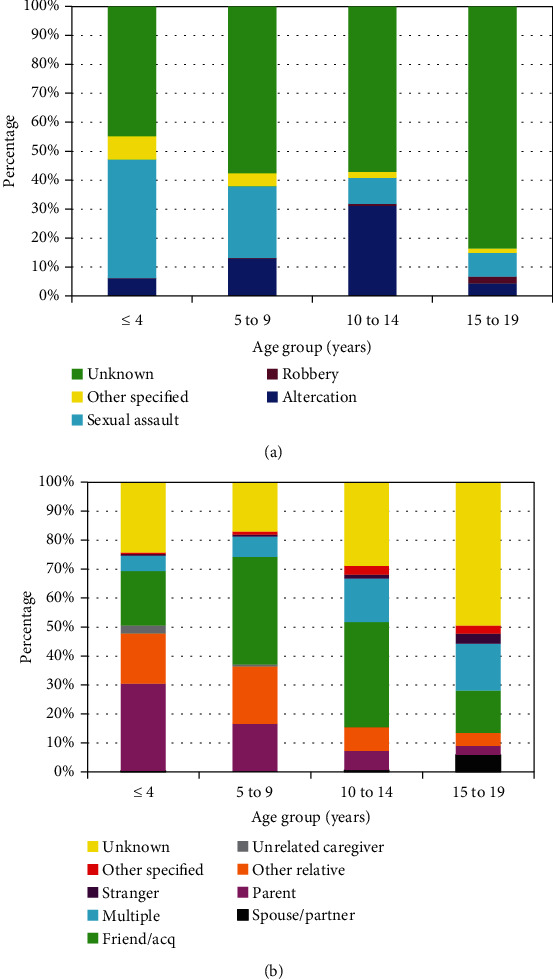
Differences by gender in child and adolescent assault victims. (a) By reason for assault (*p* < 10^−4^). (b) By perpetrator of assault (*p* < 10^−4^).

The NEISS data is divided into different age groups; for this study, the groups were ≤4 years, 5 to 9 years, 10 to 14 years, and 15 to 19 years old. With increasing age (Table 2), the percentage of sexual assaults decreased while the reason for the assault being unknown increased (Figure 2(a)). Similarly, the perpetrator being a parent decreased with increasing age (Figure 2(b)) while the identity being unknown increased. The home was the incident locale in 59.6% of those ≤ 4 years of age which decreased to 18.7% in those 15 to 19 years of age. Injuries involving the head/neck increased from 32.8% in those ≤ 4 years of age to 60.6% in those 15 to 19 years of age, while lower trunk injuries decreased from 38.9% in those ≤ 4 years to 8.1% in those 15 to 19 years old. While the vast majority of patients in all four groups were discharged from the ED, those ≤ 4 years old had the highest hospital admission rate (8.3%).

### 3.3. Analyses by Diagnosis

**Figure 3:**
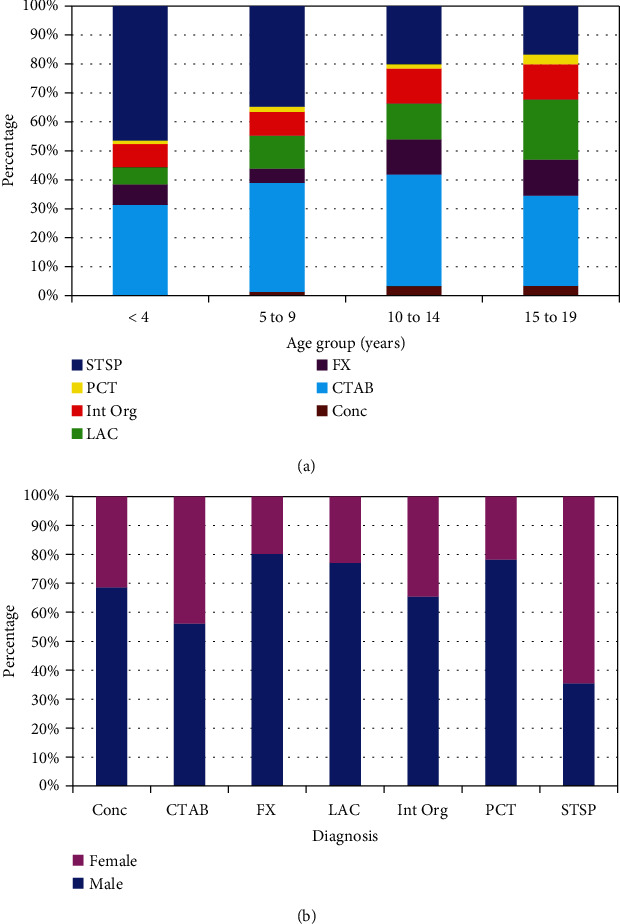
Diagnosis in child and adolescent assault victims. STSP = strain/sprain; PCT = puncture; Int Org = internal organ injury; LAC = laceration; FX = fracture; CTAB = contusion/abrasion; Conc = Concussion. (a)By age group (*p* < 10^−4^). (b) By gender (*p* < 10^−4^).

There were seven major diagnoses (Table 3) that accounted for 99.6% of all the injuries. These were concussions (*N* = 129,580—3.0%), contusion/abrasion (*N* = 1,459,483—33.3%), fracture (*N* = 504,522—11.5%), laceration (*N* = 758,519—17.3%), internal organ injury (*N* = 505,545—11.5%), puncture (*N* = 122,679—2.8%), and strain/sprain (*N* = 906,855—20.7%). The punctures and lacerations (penetrating trauma) comprised 20.1% of the assaults with blunt trauma comprising the remaining 79.9%. The common penetrating trauma was overwhelmingly in the 15- to 19-year-old age group (76.6% of the lacerations and 79.1% of the punctures were in those 15 to 19 years old). These diagnoses differed markedly by age group (Figure 3(a)) and gender (Figure 3(b)). Strain/sprains were most common in females, and fractures in males.

### 3.4. Changes Over Time

**Figure 4:**
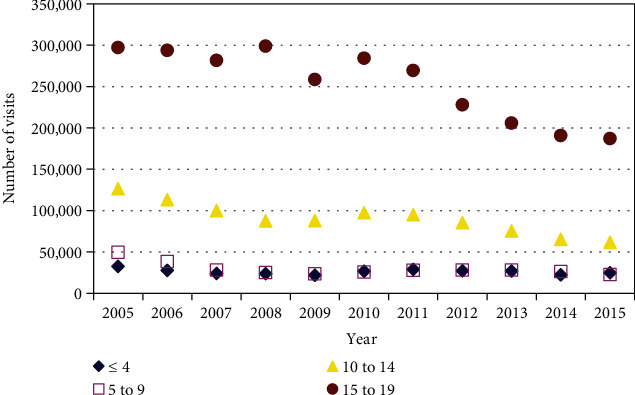
Number of assaults in child and adolescent assault victims over time and by age group. There was a gradual decrease for all age groups except for those ≤ 4 years of age. The number of assaults decreased by 23.7% for those ≤ 4 years old (*p* = 0.28, *r*^2^ = 0.13), 54.4% for those 5 to 9 years old (*p* = 0.026, *r*^2^ = 0.44), 51.1% for those 10 to 14 years old (*p* < 10^−6^, *r*^2^ = 0.84), and 37.0% for those 15 to 19 years old (*p* = 0.0007, *r*^2^ = 0.84).

There was a gradual decrease in the number of assaults from 2005 to 2015 for all age groups except for those < 4 years old (Figure 4).

## 4. Discussion

While there are some similar studies, none have focused on all injured patients who present to the emergency department across a whole country. Most focus on a certain city or county [3, 5, 6], only patients admitted to the hospital [2], or a certain type/cause of injury [7–12]. This study is more expansive, studying all assault victims in children and adolescents, not just those admitted to the hospital or having a particular type of injury, involving a particular anatomic area or encompassing a particular geographic location. In this study, only 5.0% of the patients were admitted to the hospital.

### 4.1. Literature Comparison

The study most similar to the present one is that of Barmparas et al. [2]. However, they used the National Trauma Data Bank, only studying patients admitted to the hospital. They found a slightly higher median age (16 years vs. 15 years). Both studies noted that most of the assault victims were adolescents. They noted that with increasing age, a greater proportion of their patients were Black and fewer were White. While this trend was also seen in our data until age 15 years, in those aged 15 to 19, there was an increase in the proportion of White patients (Table 2). Amerindian and Asian races demonstrated a consistent proportion across all four age groups. Barmparas et al. [2] found that younger children were more likely to sustain a head injury, while we found that they were more likely to have lower trunk injury. This difference is likely due to the fact that they only studied those admitted to the hospital. In this study, all other age groups were more likely to have a head injury, especially concussions in those 15 to 19 years of age. Additionally, the present study used different classifications for reason of injury. When known, a sexual assault was the most common reason for the assault, except for those 10 to 14 years of age, who were most likely to be injured in an altercation. This also likely explains the fact that most of the injuries in the group ≤ 4 years old involved the lower trunk (sexual assault) as discussed above. We found that adolescents were most likely to be injured in the street, while younger patients were more likely to be injured at home. Children 10 to 14 years of age were most likely to be assaulted at school and by a friend or acquaintance. This finding is consistent with other studies [6, 11] which found that older children and adolescents tended to experience more violent injury further away from home.

The study of Herbert et al. [5] from Cape Town, South Africa, had a younger population while Mollen et al. [3] studied victims of violence limited in the age from 8 to 24 years; however, both noted that most of the injuries were either to the extremities or the head. We found that in those ≤ 4 years old, most of the injuries were to the lower trunk, which has been noted by others to be more serious than other areas of injury [5].

### 4.2. Our Findings

Most patients in this study were children 15 to 19 years old (64.7%). They were more likely to be male in all age groups except those ≤ 4 years old. As has been previously noted, victims of sexual assault are more likely to be female [13]. In this study, females were more likely to be injured by a partner (8.5% vs. 0.7%) compared to males, but both sexes were more likely to be assaulted by a known person. More importantly, sexual assault accounted for 87.4% of all assaults in females (Table 1). This is likely a low estimate, as many cases of sexual assault are not reported to health care providers [14–18] or police [19]. The perpetrator was unknown in 45.2% of male and 33.5% of female victims (Table 1).

All of the diagnoses (concussions, contusions/abrasions, fractures, lacerations, internal organ injuries, and punctures) were most likely to occur in 15- to 19-year-olds and male patients and least likely to occur in the younger patients. Strains and sprains were the only injuries to occur more often in female patients and have a younger average age of injury presentation. This finding differs from Mollen et al. [3] who found that females were more likely to sustain bruises/abrasions and be injured in an event involving multiple perpetrators. They also noted that older patients were less likely to sustain a fracture [3]. This information may coincide with results from a study of Indianapolis youth [6] which found that there was a significant spike in violent injury events between the ages of 13 and 16. This is in contrast to Mollen et al. [3] who found that females were more likely to sustain bruises/abrasions and be injured by multiple perpetrators. They also found that older patients were less likely to sustain a fracture. The discrepancies between this study and that of Mollen et al. [3] are likely due to the fact that their study was limited to the Philadelphia area, thus not representative of the entire US, as well as limiting the patient age from 8 to 24 years.

Although concussions only accounted for 3.0% of the diagnoses, nearly all occurred in those ≥ 10 years of age. This is likely due to the fact that those ≥ 10 years old also accounted for nearly all of the altercations. Altercations often involve fighting, where exchanges of blows are likely to result in a concussion if delivered to the head. Within the youngest age group (≤ 4 years old), strains/sprains were quite common. These are typically less severe injuries than fractures, concussions, and internal organ injuries. The exact reason why there are more strains/sprains in this age group cannot be stated with certainty. Possible explanations are that, in spite of parents being the most common perpetrator of the assault of the four different age groups, the assault involved lower amounts of energy being delivered to the patient, resulting in a strain/sprain. Also, if the perpetrator was another child, such as in a day care center, a younger child would also likely not be able to deliver adequate injury that would result in a fracture or concussion. However, nearly 50% of the children in the ≤4-year-old age group were assaulted by parents or other relatives. These could also be defined as child abuse, battered child syndrome, or nonaccidental trauma. This leads to the next topic.

### 4.3. Government and Other Social Factors

All 50 of the states in the US have mandatory reporting of potential or actual child abuse to appropriate legal authorities for certain professionals and groups [20]. These include social workers, teachers/other school personnel, all health care workers, counselors and mental health professions, child care providers, and law enforcement officers. Also, anyone can file a concern for child maltreatment with appropriate authorities, and in 18 states, it is law that any person who suspects child abuse or neglect is required to report such concerns [20]. Most states have a toll-free number to call to report suspected abuse. Child Welfare Information Gateway, a service of the Children's Bureau (https://www.childwelfare.gov), provides a list of state child abuse reporting numbers. Another source on how and where to file a report of suspected child abuse and neglect is the National Child Abuse Hotline and can be reached 7 days a week, 24 hours a day, at its toll-free number, 1.800.4-A-CHILD (1.800.422.4453).

Once such a report has been filed, then each state's Child Protective Services agency follows its own investigation algorithm. The Child Protective Services response is often differential [21]. In serious cases, the state will take legal custody of the child and place them into foster care. In less serious cases, they will use community agencies to support families who are considered lower risk, recognizing that variations in families' needs and strengths require different approaches. In-home services play an important role in safety and permanence for the majority of families that receive a report of child maltreatment [22].

There is now an even stronger push to keep children in their own home when possible. The 2018 signing of the Family First Prevention Services Act (H.R. 1892) [23] redirects federal funds to provide services to keep children safely with their families and out of foster care, and when foster care is needed, it allows federal reimbursement for care in family-based settings and certain residential treatment programs for children with emotional and behavioral disturbance requiring special treatment. As the data used in this was collected before the implementation of this law, further research and follow-up will be needed to assess its impact on the incidence of child maltreatment occurring in their own home.

### 4.4. Limitations

There are certain limitations to this study. One potential limitation is the accuracy of the NEISS data. However, two studies have demonstrated over 90% accuracy [24, 25]. The NEISS only identifies individuals who sought care in an ED. It does not include those who might have been treated in urgent care centers, physician offices, and other non-ED venues or those persons who did not seek medical care, and therefore, the assault was never reported to any agency collecting such data. Another limitation is injury severity. The only proxy of injury severity with NEISS data is disposition from the ED as being treated and released or admitted to the hospital. The NEISS-AIP does not include fatal injuries nor does it record the Injury Severity Score. Finally, the race was not known in 20.4% of the patients; this is due to either the patient refusing to divulge such information or it not being collected on the medical record so that the NEISS coders could include it. However, acknowledging these limitations, we noted many interesting findings as described above.

## 5. Conclusion

These data provide a comprehensive overview of child and adolescent assault victims presenting to the ED in the USA. They can be used as background data for further study. The decreasing numbers of assaults over the 11 years of the study are encouraging, but there still exist challenges in decreasing the number for those ≤ 4 years old.

## Figures and Tables

**Table 1:** General demographics and demographics by gender.

	All					Male					Female					% female	*p* value
	*n*	*N*	U95%	L95%	%	*n*	*N*	U95%	L95%	%	*n*	*N*	U95%	L95%	%		
Average age ± 1 SD (years)	14.5 ± 5.1					14.8 ± 4.8					14.0 ± 5.4						<10^−4^
Age group (years)																	
≤4	10,017	286,857	359,382	228,298	6.5	4,176	119,739	146,910	97,411	4.5	5,841	167,118	210,389	132,084	9.5	58.3	<10^−4^
5 to 9	9,748	322,079	366,420	282,843	7.3	5,287	183,605	212,821	158,027	6.9	4,461	138,475	157,135	121,749	7.9	43.0	
10 to 14	25,031	995,786	1,072,429	922,869	22.6	14,658	604,745	651,433	560,375	22.8	10,373	391,041	426,733	357,538	22.3	39.3	
15 to 19	57,749	2,794,082	2,892,214	2,692,948	63.5	35,430	1,738,937	1,798,125	1,677,950	65.7	22,319	1,055,145	1,099,767	1,009,550	60.2	37.8	
Race																	
White	27,090	1,517,881	2,089,390	992,977	43.3	15,349	903,556	1,260,861	579,068	42.7	11,741	614,325	830,312	412,866	44.3	40.5	0.015
Black	40,032	1,264,082	1,592,551	974,043	36.1	23,349	751,445	955,018	570,173	35.5	16,683	512,636	635,124	401,204	36.9	40.6	
Amerindian	12,877	692,488	1,235,612	350,978	19.7	7,853	440,438	779,645	224,087	20.8	5,024	252,050	455,485	127,025	18.2	36.4	
Asian	699	31,826	59,957	16,830	0.9	471	22,587	42,784	11,861	1.1	228	9,239	17,492	4,859	0.7	29.0	
Reason																	
Altercation	27,268	1,244,983	1,331,674	1,161,579	28.3	17,564	814,419	878,188	753,188	30.7	9,704	430,565	484,961	380,327	24.6	34.6	<10^−4^
Robbery/burglary	1,253	53,672	73,150	39,219	1.2	1,024	42,806	56,529	32,378	1.6	229	10,865	17,702	6,660	0.6	20.2	
Sexual assault	14,810	453,496	583,433	349,884	10.3	2,275	57,234	77,760	41,932	2.2	12,535	396,262	488,116	317,407	22.6	87.4	
Other specified	2,677	87,088	103,114	73,590	2.0	1,607	53,934	66,348	43,790	2.0	1,070	33,154	37,857	28,919	1.9	38.1	
Unknown	56,551	2,558,422	2,685,821	2,428,916	58.1	37,117	1,678,462	1,747,618	1,607,225	63.2	19,434	879,960	936,972	822,873	50.2	34.4	
Perpetrator																	
Spouse/partner	3,190	168,516	189,043	150,265	3.8	358	18,912	22,824	15,658	0.7	2,832	149,604	164,575	135,831	8.5	88.8	<10^−4^
Parent	8,245	293,505	333,579	257,786	6.7	3,672	132,079	151,540	114,916	5.0	4,573	161,426	179,823	144,770	9.2	55.0	
Other relative	8,660	319,136	350,324	290,835	7.2	3,878	151,728	168,790	136,412	5.7	4,782	167,408	182,101	153,883	9.6	52.5	
Friend/acquaintance	22,655	942,928	1,001,619	886,607	21.4	12,854	568,282	611,202	527,603	21.4	9,801	374,646	394,173	355,790	21.4	39.7	
Multiple	15,281	642,918	763,663	538,486	14.6	9,346	409,989	490,713	340,500	15.4	5,935	232,930	273,415	197,700	13.3	36.2	
Stranger	2,883	113,461	140,570	91,657	2.6	2,023	81,162	99,788	65,818	3.1	860	32,298	41,012	25,414	1.8	28.5	
Other specified	2,303	110,746	120,741	101,792	2.5	1,780	86,635	96,073	78,026	3.3	523	24,111	26,816	21,733	1.4	21.8	
Unknown	39,072	1,801,229	1,911,582	1,693,015	40.9	25,616	1,198,517	1,272,563	1,125,270	45.2	13,456	602,712	644,803	561,902	34.4	33.5	
Incident locale																	
Unknown	36,784	1,620,207	1,930,530	1,332,996	36.8	22,069	993,134	1,195,334	806,266	37.4	14,715	627,073	739,096	523,344	35.8	38.7	<10^−4^
Home	26,586	1,019,068	1,204,764	854,439	23.1	11,136	449,374	540,076	371,286	16.9	15,450	569,694	659,701	486,538	32.5	55.9	
School/sports	19,195	862,116	943,893	786,137	19.6	12,680	585,871	641,457	533,707	22.1	6,515	276,246	309,520	245,898	15.8	32.0	
Street	9,485	440,176	621,330	307,580	10.0	6,814	312,618	438,165	219,481	11.8	2,671	127,558	186,483	86,231	7.3	29.0	
Other property	10,708	464,533	581,671	368,832	10.5	7,045	312,590	399,683	242,570	11.8	3,663	151,943	185,607	123,913	8.7	32.7	
Anatomic location																	
Unknown	2,572	87,316	137,045	55,523	2.0	781	25,706	39,278	16,720	1.0	1,791	61,610	100,953	37,156	3.5	70.6	<10^−4^
Head/neck	54,620	2,489,646	2,583,147	2,395,426	56.5	35,868	1,654,719	1,690,028	1,614,125	62.3	18,752	834,927	899,289	770,995	47.6	33.5	
Upper trunk	6,976	308,217	331,817	285,988	7.0	4,521	199,149	218,950	180,999	7.5	2,455	109,068	117,954	100,778	6.2	35.4	
Lower trunk	15,961	520,630	642,923	419,067	11.8	4,411	153,453	178,079	131,901	5.8	11,550	367,177	459,722	289,013	20.9	70.5	
Upper extremity	15,442	700,595	743,834	659,227	15.9	10,316	471,143	643,845	598,728	17.8	5,126	229,451	255,012	206,113	13.1	32.8	
Lower extremity	7,197	300,192	341,511	263,515	6.8	3,851	149,766	172,241	130,043	5.6	3,346	150,426	179,472	125,666	8.6	50.1	
Disposition from ED																	
Release	94,251	4,110,626	4,186,797	3,997,177	95.0	53,657	2,441,560	2,498,849	2,358,485	93.4	40,594	1,669,065	1,687,753	1,638,350	97.3	40.6	<10^−4^
Admit	6,712	218,603	332,052	142,432	5.0	5,211	172,298	255,374	115,010	6.6	1,501	46,305	77,020	27,617	2.7	21.2	
Hospital size																	
Small	6,408	779,276	1,096,361	538,927	17.7	3,647	443,477	634,291	301,487	16.7	2,761	335,799	464,104	236,434	19.2	43.1	0.012
Medium	6,966	887,698	1,234,288	619,568	20.1	4,260	539,925	756,106	373,409	20.3	2,706	347,773	486,363	241,166	19.8	39.2	
Large	17,353	1,535,598	2,189,197	990,162	34.8	10,910	965,666	1,386,151	611,201	36.4	6,443	569,932	804,646	376,471	32.5	37.1	
Very large	41,098	937,644	1,302,590	653,498	21.3	24,827	566,153	802,816	384,821	21.3	16,271	371,490	504,766	265,878	21.2	39.6	
Children's	30,943	266,381	479,878	144,536	6.0	16,104	138,716	253,982	74,045	5.2	14,839	127,665	227,495	69,581	7.3	47.9	

*n* = actual number of ED visits; *N* = estimated number of ED visits; L95% = lower 95% confidence interval of the estimate; U95% = upper 95% confidence interval of the estimate. Those categories comprising less than 1% of the variables as described in the appendix are excluded; thus, the percentage sum will not add up to 100.

**Table 2:** Demographics by age group.

	≤4 years					5 to 9 years					10 to 14 years					15 to 19 years				%	*p* value
	*n*	*N*	L95%	U95%	%	*n*	*N*	L95%	U95%	%	*n*	*N*	L95%	U95%	%	*n*	*N*	L95%	U95%		
Sex																					
Male	4,176	119,739	111,042	128,627	41.7	5,287	183,605	170,316	196,598	57.0	14,658	604,775	592,393	616,889	60.7	35,430	1,738,937	1,681,758	1,794,918	62.2	<10^−4^
Female	5,841	167,118	158,230	175,815	58.3	4,461	138,475	125,482	151,764	43.0	10,373	391,041	378,897	403,393	39.3	22,319	1,055,145	999,164	1,112,324	37.8	
Race																					
White	3,652	120,734	85,764	153,782	54.6	2,725	106,686	61,341	158,418	41.5	5,943	320,890	196,764	463,823	39.8	14,749	968,890	647,627	1,316,096	43.7	<10^−4^
Black	2,790	58,623	39,541	82,732	26.5	3,735	90,703	64,761	120,523	35.3	10,987	315,873	241,475	397,159	39.1	22,432	796,637	616,366	996,827	35.9	
Amerindian	1,184	40,819	23,123	67,443	18.4	1,270	56,185	28,640	98,799	21.9	3,187	164,481	78,689	304,750	20.4	7,220	430,248	217,945	769,792	19.4	
Asian	58	1,093	553	2,146	0.5	88	3,515	1,800	6,787	0.0	154	5,627	3,067	10,976	0.7	398	21,369	10,864	41,904	1.0	
Reason																					
Altercation	533	17,315	13,024	22,893	6.0	1,187	41,309	36,694	46,424	12.8	7,681	308,946	281,813	336,583	31.0	17,853	87,678	819,286	936,367	3.1	<10^−4^
Robbery/burglary	12	338	172	631	0.1	18	616	226	1,675	0.2	198	7,055	4,182	11,950	0.7	1,016	45,392	33,811	60,916	1.6	
Sexual assault	4,441	117,669	99,061	137,216	41.0	3,240	80,435	61,598	102,770	25.0	3,143	89,755	69,607	115,016	9.0	3,986	165,624	125,743	216,837	5.9	
Other specified	957	23,181	19,910	26,967	8.1	490	14,286	11,888	17,139	4.4	578	20,045	16,530	24,198	2.0	651	29,555	22,913	38,282	1.1	
Unknown	4,075	128,333	111,970	145,105	44.7	4,812	185,502	166,752	203,608	57.6	13,395	568,737	537,337	599,575	57.1	34,078	1,669,333	1,590,230	1,746,711	59.7	
Perpetrator																					
Spouse/partner	17	809	430	1,549	0.3	6	68	32	161	0.0	157	5,368	3,983	7,170	0.5	3,007	162,203	144,465	181,908	5.8	<10^−4^
Parent	2,933	86,586	80,872	92,520	30.2	1,616	53,295	43,589	64,658	16.5	1,829	66,290	57,956	75,681	6.7	1,867	87,334	76,005	100,315	3.1	
Other relative	1,905	49,724	44,696	55,168	17.3	2,115	63,766	58,054	69,877	19.8	2,046	81,267	72,395	91,116	8.2	2,595	124,378	112,330	137,758	4.5	
Unrelated caregiver	302	7,818	6,197	9,869	2.7	73	2,302	1,546	3,447	0.7	44	20	996	2,490	0.0	35	1,468	838	2,235	0.1	
Friend/acquaintance	1,955	53,864	49,946	58,008	18.8	3,504	119,545	105,799	133,956	37.1	8,756	360,802	344,250	377,809	36.2	8,436	408,563	354,037	470,000	14.6	
Multiple	629	15,486	13,168	18,188	5.4	737	22,615	20,361	25,097	7.0	4,080	150,827	120,791	186,714	15.1	9,823	453,753	381,141	537,342	16.2	
Stranger	43	1,234	832	1,807	0.4	55	2,064	1,450	2,932	0.6	415	12,433	10,058	15,335	1.2	2,352	97,008	77,402	121,552	3.5	
Other specified	37	1,649	1,148	2,381	0.6	95	3,297	2,513	4,317	1.0	728	30,247	26,688	34,256	3.0	1,423	75,552	68,181	83,549	2.7	
Unknown	2,179	69,713	63,258	76,598	24.3	1,546	55,083	49,001	61,727	17.1	6,969	286,770	259,607	315,571	28.8	28,199	1,383,384	1,316,949	1,449,957	49.5	
Incident locale																					
Unknown	2,542	84,218	69,254	100,925	29.4	2,218	74,272	58,054	93,428	23.1	7,268	270,454	220,273	327,222	27.2	24,653	1,187,179	955,647	1,431,235	42.5	<10^−4^
Home	6,445	170,958	155,749	182,687	59.6	4,411	131,254	109,987	153,640	40.7	5,358	193,660	161,022	231,027	19.4	10,356	522,617	426,688	634,304	18.7	
School/sports	671	19,845	15,262	25,676	6.9	2,546	95,004	70,747	123,518	29.5	8,579	377,087	347,935	407,185	37.9	7,399	370,264	314,916	433,394	13.3	
Street	112	3,725	2,238	6,168	1.3	260	9,457	7,216	12,339	2.9	1,690	77,337	51,882	113,721	7.8	7,366	348,235	243,383	489,560	12.5	
Other property	248	8,107	6,053	10,844	2.8	313	12,168	8,698	16,978	3.8	2,134	77,150	60,645	97,689	7.7	7,974	365,657	289,488	458,264	13.1	
Anatomic location																					
Head/neck	3,043	93,972	78,950	110,364	32.8	3,607	131,804	115,013	149,259	40.9	13,852	567,114	542,914	591,908	57.0	34,026	1,693,398	1,643,043	1,742,799	60.6	<10^−4^
Upper trunk	458	13,063	10,902	15,635	4.6	588	21,683	19,491	24,130	6.7	1,501	65,076	59,748	70,901	6.5	4,382	206,583	189,173	225,220	7.4	
Lower trunk	4,317	111,495	91,430	132,970	38.9	3,251	84,128	66,398	104,639	26.1	3,213	98,207	78,967	121,488	9.9	5,136	225,432	184,144	274,958	8.1	
Upper extremity	586	16,650	13,971	19,795	5.8	1,036	37,670	33,956	41,752	11.7	4,438	184,055	172,673	195,975	18.5	9,374	461,971	432,556	492,913	16.5	
Lower extremity	17,168	33,846	27,971	40,766	11.8	816	33,390	27,674	40,142	10.4	1,501	64,013	56,064	72,993	6.4	3,811	168,324	145,583	194,483	6.0	
Disposition from ED																					
Release	8,736	259,819	244,976	269,225	91.7	9,409	312,693	306,250	315,844	98.1	23,984	963,933	945,877	973,868	97.8	52,093	2,573,306	2,493,535	2,627,796	94.1	0.0005
Admit	1,142	23,456	14,050	38,299	8.3	207	6,019	2,868	12,462	1.9	780	21,665	11,729	39,720	2.2	4,408	161,133	106,643	240,904	5.9	
Hospital size																					
Small	534	65,216	44,610	91,745	22.7	535	65,156	41,205	98,196	20.2	1,495	181,770	120,493	264,686	18.3	3,843	467,014	325,814	653,305	16.7	<10^−4^
Medium	237	31,526	19,795	48,942	11.0	336	43,526	27,030	67,816	13.5	1,504	193,179	125,372	285,597	19.4	4,889	619,468	440,380	844,993	22.2	
Large	1,022	90,183	57,836	130,331	31.4	1,272	111,658	60,148	177,416	34.7	3,856	340,230	195,875	285,597	34.2	11,165	990,152	655,261	1,385,409	35.4	
Very large	2,039	46,684	27,684	74,963	16.3	2,557	58,396	34,021	94,491	18.1	8,722	199,260	121,488	521,604	20.0	27,610	629,400	455,469	845,832	22.5	
Children's	6,188	53,274	27,311	94,930	18.6	5,049	43,428	22,713	78,093	13.5	9,455	81,368	43,517	309,198	8.2	10,245	88,255	47,503	162,069	3.2	

*n* = actual number of ED visits; *N* = estimated number of ED visits; L95% = lower 95% confidence interval of the estimate; U95% = upper 95% confidence interval of the estimate. Those categories comprising less than 1% of the variables as described in the appendix are excluded; thus, the percentage sum will not add up to 100.

**Table 3:** Demographics by major diagnoses.

	Concussion					Contusion/abrasion					Fracture					Laceration									Internal organ						Puncture		Strain/sprain	*p* value		
	*n*	*N*	L95%	U95%	%	*n*	*N*	L95%	U95%	%	*n*	*N*	L95%	U95%	%	*n*	*N*	L95%	U95%	%	*n*	*N*	L95%	U95%	%	*n*	*N*	L95%	U95%	%	*n*	*N*	L95%	U95%	%	
Average age ± 1 SD (years)	15.7 ± 3.1					14.2 ± 4.9					15.2 ± 4.8					15.8 ± 3.9					14.9 ± 4.7					15.7 ± 4.1					12.8 ± 3.4			<10^−4^		
Age group (years)																																				<10^−4^
≤4	21	521	259	1,048	0.4	2,593	86,948	69,164	108,853	6.0	874	19,635	13,197	29,013	3.9	491	16,550	13,414	20,463	2.2	774	22,313	16,894	29,300	4.4	69	3,450	1,466	7,932	2.9	4,886	129,464	102,769	161,714	14.3	
5 to 9	141	4,052	1,113	2,407	3.1	3,037	120,383	105,934	136,576	8.3	506	15,804	12,089	20,601	3.1	1,030	36,397	30,391	43,578	4.8	735	26,130	20,929	32,578	5.2	118	5,793	2,452	13,171	4.8	4,101	111,056	98,876	124,500	12.3	
10 to 14	977	32,386	28,653	36,431	25.0	8,513	382,981	344,797	423,737	26.2	2,983	119,891	109,051	131,465	23.8	3,170	123,542	113,531	134,524	16.3	2,898	118,838	107,770	130,665	23.6	395	15,928	11,669	21,439	13.3	6,020	199,344	184,713	214,864	22.0	
15 to 19	2,055	92,459	87,784	96,805	71.4	16,556	868,838	823,252	913,282	59.5	7,080	348,368	336,168	360,094	69.2	11,961	580,629	566,895	594,709	76.6	6,882	337,023	323,713	349,836	66.8	2,468	95,005	80,830	105,034	79.1	10,595	465,591	430,544	500,445	51.4	
Sex																																				
Male	2,209	88,863	85,458	92,118	68.6	17,187	817,807	799,437	836,067	56.0	9,003	404,347	397,859	410,522	80.1	12,584	584,098	574,357	593,543	77.0	7,258	330,499	319,044	341,591	65.4	2,493	95,855	85,385	104,032	78.1	8,676	321,102	278,833	365,974	35.4	<10^−4^
Female	992	40,717	37,462	44,122	31.4	13,521	641,552	623,293	659,923	44.0	2,463	100,166	93,991	106,654	19.9	4,107	174,229	164,784	183,970	23.0	4,056	175,037	163,945	186,492	34.6	622	26,824	18,647	37,294	21.9	16,973	585,673	540,801	627,942	64.6	
Race																																				
White	1,050	61,213	49,560	71,955	59.1	8,050	509,297	323,501	714,234	42.6	3,268	191,941	134,381	250,850	48.0	3,571	217,560	129,901	324,845	35.3	3,133	181,413	118,947	244,972	48.7	327	15,093	8,837	24,497	16.1	7,541	335,598	224,197	451,099	47.1	<10^−4^
Black	1,055	25,423	16,713	36,749	24.6	12,568	432,994	338,667	537,019	36.3	4,202	131,405	99,596	167,727	32.9	7,469	258,985	205,292	315,976	42.0	3,257	107,107	76,181	144,502	28.8	1,606	51,090	31,864	68,961	54.6	9,629	250,810	183,117	328,001	35.2	
Amerindian	348	15,352	9,262	24,413	14.8	4,082	238,734	110,819	452,710	20.0	1,434	73,738	38,143	130,543	18.4	2,241	132,636	65,844	237,813	21.5	1,537	80,809	82,477	141,447	21.7	391	26,795	13,068	46,598	28.6	2,771	121,803	62,083	219,569	17.1	
Asian	32	1,500	704	3,156	1.4	241	13,146	6,329	27,347	1.1	68	2,741	1,399	5,358	0.7	134	6,756	3,388	13,304	1.1	83	3,195	1,714	5,960	0.9	15	630	300	1,320	0.7	119	3,751	2,278	6,052	0.5	
Reason																																				
Altercation	848	31,990	28,080	36,256	24.7	8,723	433,742	401,212	467,764	29.7	4,422	200,255	181,833	219,047	39.7	6,014	267,732	243,181	293,319	35.3	2,797	117,918	108,136	128,307	23.3	517	22,389	16,402	29,934	18.3	3,875	168,912	146,004	194,520	18.6	<10^−4^
Robbery/burglary	52	1,361	907	1,944	1.1	341	15,701	11,092	22,038	1.1	152	5,910	4,286	8,118	1.2	307	14,770	9,937	21,845	1.9	188	7,810	5,460	11,173	1.5	67	2,335	1,779	3,055	1.9	142	5,464	3,990	7,436	0.6	
Sexual assault	4	109	26	518	0.1	434	14,988	10,508	21,454	1.0	19	542	252	1,160	0.1	111	4,397	2,579	7,509	0.6	24	685	354	1,264	0.1	3	104	25	491	0.1	14,212	432,545	364,102	501,944	47.7	
Other specified	34	1,050	505	2,164	0.8	1,185	39,257	32,109	47,871	2.7	3,334	9,620	7,009	13,111	1.9	180	7,523	5,992	9,406	1.0	242	8,247	6,926	9,808	1.6	126	5,225	2,760	9,692	4.3	480	14,014	11,154	17,593	1.5	
Unknown	2,253	94,483	89,475	99,077	72.9	19,984	954,054	918,015	988,946	65.4	6,512	287,307	269,977	304,014	57.0	10,038	461,959	438,196	484,997	60.9	8,031	369,375	357,471	380,625	73.1	2,374	91,294	85,838	96,193	74.4	6,924	285,213	236,054	339,345	31.5	
Perpetrator																																				
Spouse/partner	76	3,703	2,786	4,898	2.9	1,336	75,153	64,655	87,277	5.1	2,221	11,350	9,177	13,968	2.3	455	22,299	18,660	26,548	2.9	365	18,710	15,824	22,092	3.7	57	3,265	2,306	4,600	2.7	671	33,651	28,385	39,902	3.7	<10^−4^
Parent	87	3,639	2,799	4,717	2.8	3,704	146,722	125,661	170,760	10.1	446	14,696	11,850	18,153	2.9	526	20,384	17,673	23,514	2.7	641	24,273	19,868	29,574	4.8	35	1,567	920	2,662	1.3	2,620	77,860	67,379	89,779	8.6	
Other relative	116	4,948	3,499	6,958	3.8	2,055	98,017	86,255	111,213	6.7	656	25,953	22,389	30,003	5.1	1,490	59,888	53,020	67,584	7.9	606	23,630	20,272	27,502	4.7	99	3,909	2,110	7,140	3.2	3,542	100,251	89,235	112,359	11.1	
Friend/acquaintance	869	32,355	29,285	35,635	25.0	6,835	324,462	308,681	340,789	22.2	2,232	97,439	87,891	107,658	19.3	3,143	135,055	117,798	154,207	17.8	2,477	112,084	101,766	123,151	22.2	350	16,400	9,385	27,382	13.4	6,678	222,728	206,400	239,863	24.6	
Multiple	816	28,528	24,231	33,354	22.0	5,142	232,283	184,625	289,415	15.9	1,548	62,270	56,930	80,075	12.3	2,210	101,729	75,852	134,637	13.4	2,782	114,150	99,390	130,431	22.6	184	8,192	6,784	9,876	6.7	2,548	22,857	79,531	98,031	2.5	
Stranger	75	2,243	1,697	2,954	1.7	464	20,543	16,638	25,249	1.4	294	11,392	8,421	15,329	2.3	506	22,978	19,039	27,686	3.0	284	13,024	10,414	16,329	2.6	445	15,212	10,084	22,426	12.4	806	27,509	19,951	37,725	3.0	
Other specified	7	180	65	505	0.1	576	31,207	27,000	36,049	2.1	1,030	46,843	41,853	52,291	9.3	203	10,145	8,192	12,591	1.3	32	1,476	1,011	2,174	0.3	22	1,634	761	2,429	1.3	427	19,331	15,689	23,760	2.1	
Unknown	1,154	53,975	48,955	59,140	41.7	10,408	525,182	474,478	578,101	36.0	5,002	228,203	215,366	240,881	45.3	8,147	385,365	363,027	407,704	50.8	4,095	197,224	181,541	213,340	39.0	1,917	72,664	60,419	84,047	59.2	8,178	331,872	302,980	361,926	36.6	
Incident locale																																				
Unknown	973	37,540	27,924	48,865	29.0	10,090	486,378	396,979	584,669	33.3	4,781	206,266	168,773	245,772	40.9	6,703	303,689	238,023	374,481	40.0	4,403	205,820	163,797	250,700	40.7	1,009	41,077	28,179	56,359	33.5	8,684	334,006	281,306	390,084	36.8	<10^−4^
Home	404	18,581	15,550	22,080	14.3	8,040	360,952	300,799	428,650	24.7	2,018	83,889	69,133	100,951	16.6	3,071	139,087	109,378	174,687	18.3	1,909	73,122	56,722	93,222	14.5	454	20,018	16,721	23,824	16.3	10,299	312,898	265,981	363,467	34.5	
School/sports	1,070	40,060	36,697	43,578	30.9	6,756	327,984	297,589	360,492	22.5	2,387	108,056	96,161	120,869	21.4	2,871	127,793	109,758	148,139	16.8	2,669	119,812	106,771	133,919	23.7	297	14,400	7,557	26,032	11.7	3,114	122,514	109,004	137,389	13.5	
Street	326	16,993	10,587	26,408	13.1	2,660	142,208	99,245	200,971	9.7	1,045	49,317	32,877	72,612	9.8	2,005	97,382	67,356	139,264	12.8	880	43,006	28,007	64,963	8.5	1,016	32,164	19,665	48,839	26.2	1,535	57,788	37,634	87,693	6.4	
Other property	428	16,407	13,697	19,567	12.7	3,162	141,780	110,483	180,684	9.7	1,236	15,664	45,937	70,242	3.1	2,044	90,119	69,860	115,219	11.9	1,453	63,776	47,218	85,083	12.6	339	15,019	10,845	20,512	12.2	2,016	79,372	63,661	98,484	8.8	
Anatomic location																																				
Unknown	0	0	0	0	0.0	772	28,408	14,449	55,606	1.9	6	188	50	706	0.0	20	668	303	1,593	0.1	0	0	0	0	0.0	11	282	123	675	0.2	1,758	57,573	35,005	93,134	6.3	<10^−4^
Head/neck	3,201	129,580	5,183	8,773	100.0	17,788	864,682	824,024	904,442	59.2	5,583	264,463	253,135	275,473	52.4	12,642	573,946	559,559	587,549	75.7	10,948	491,673	485,475	495,990	97.3	408	17,648	12,317	24,781	14.4	3,890	142,121	124,602	161,602	15.7	
Upper trunk	0	0	0	0	0.0	3,518	165,040	149,743	181,706	11.3	405	15,027	11,900	18,909	3.0	567	24,961	19,873	31,251	3.3	219	8,024	5,308	12,083	1.6	686	27,184	21,677	33,626	22.2	1,514	66,079	53,504	81,345	7.3	
Lower trunk	0	0	0	0	0.0	2,132	99,615	82,023	120,699	6.8	94	3,782	2,773	5,143	0.8	338	14,859	12,212	18,053	2.0	148	5,848	3,792	8,999	1.2	563	21,602	16,145	28,412	17.6	12,596	372,305	305,338	443,180	41.1	
Upper extremity	0	0	0	0	0.0	4,557	220,084	206,079	234,977	15.1	4,553	193,522	180,018	207,197	38.4	2,637	121,514	112,792	130,769	16.0	0	0	0	0	0.0	663	28,567	20,328	38,877	23.3	2,871	130,726	113,810	149,631	14.4	
Lower extremity	0	0	0	0	0.0	1,943	81,653	74,288	89,758	5.6	826	27,540	21,733	34,793	5.5	490	22,571	19,873	25,638	3.0	0	0	0	0	0.0	784	27,396	22,720	32,718	22.3	3,026	138,051	109,095	173,028	15.2	
Disposition from ED																																				
Release	2,909	122,178	116,361	125,441	94.6	29,616	1,411,193	1,386,136	1,423,273	98.4	9,872	456,900	437,159	470,879	91.3	15,821	722,625	713,651	728,787	97.4	9,952	460,606	436,482	475,624	92.6	1,858	80,187	61,549	95,415	66.3	23,717	839,692	805,339	859,951	94.7	<10^−4^
Admit	281	6,984	3,720	12,800	5.4	591	22,691	10,611	47,748	1.6	1,501	43,395	29,417	63,137	8.7	512	19,370	13,208	28,344	2.6	1,175	36,752	21,735	60,877	7.4	1,214	40,805	25,578	59,444	33.7	1,341	46,856	26,596	81,208	5.3	
Hospital size																																				
Small	196	24,037	16,936	33,237	18.5	2,505	303,948	203,890	435,948	20.8	727	88,418	62,628	121,777	17.5	950	115,360	72,894	176,128	15.2	447	54,788	29,372	97,975	10.8	76	9,216	4,932	16,697	7.5	1,472	179,271	125,237	249,204	19.8	<10^−4^
Medium	259	30,923	20,422	44,614	23.9	2,680	337,993	228,847	478,856	23.2	867	109,777	79,067	148,099	21.8	1,236	157,013	100,959	233,093	20.7	724	96,528	62,890	142,361	19.1	97	12,077	5,717	24,045	9.8	1,079	140,281	89,325	212,748	15.5	
Large	457	40,610	29,739	53,335	31.3	5,333	470,519	254,826	754,407	32.2	1,984	175,824	124,248	235,334	34.9	3,188	281,850	147,684	448,436	37.2	2,497	222,527	165,010	283,459	44.0	600	52,913	26,805	82,563	43.1	3,234	286,070	191,165	401,555	31.5	
Very large	1,004	22,977	15,498	33,017	17.7	12,193	278,097	181,852	408,947	19.1	4,404	100,428	73,419	134,131	19.9	7,547	171,618	105,662	262,220	22.6	4,624	10,582	68,704	155,961	2.1	2,014	45,606	24,548	72,172	37.2	9,132	208,948	143,011	293,549	23.0	
Children's	1,285	11,035	6,336	18,685	8.5	7,999	68,927	37,217	125,078	4.7	3,485	30,075	15,985	55,064	6.0	3,773	32,678	15,246	68,115	4.3	3,023	25,830	15,318	42,921	5.1	328	2,866	1,141	7,079	2.3	10,738	92,284	46,612	173,753	10.2	

*n* = actual number of ED visits; *N* = estimated number of ED visits; L95% = lower 95% confidence interval of the estimate; U95% = upper 95% confidence interval of the estimate. Those categories comprising less than 1% of the variables as described in the appendix are excluded; thus, the percentage sum will not add up to 100.

## Data Availability

The raw data is in the public domain and housed by the Inter-University Consortium for Political and Social Research (ICPSR). It can be accessed at https://www.icpsr.umich.edu/icpsrweb/ICPSR/search/studies?q=all+injury+program. The refined data are available from the corresponding author upon request.

## Appendix

### A. NEISS Definitions [26]

#### A.1 Assault

Assault is defined as injury from an act of violence where physical force by one or more persons is used with the intent of causing harm, injury, or death to another person or an intentional poisoning by another person. This category includes perpetrators as well as intended and unintended victims of violent acts (e.g., innocent bystanders). This category excludes unintentional shooting victims (other than those occurring during an act of violence), unintentional drug overdoses, and children or teenagers “horsing” around.

#### A.2 Hospital Strata

Four are based on size (the total number of ED visits reported by the hospital, which are small (0–16,830), medium (16,831–21,850), large (28,151–41,130), and very large (>41,130)), and one includes children's hospitals of all sizes. The actual age is also categorized into 18 different groups in 5-year increments with the last group including all those ≥ 85 years old. The injured body part is classified into five major locations (head/neck, upper trunk, lower trunk, upper extremity, and lower extremity).

#### A.3 Incident Locale

This is categorized into home/apartment/mobile, school/sports, street, other property, farm, and unknown. Other property consists of stores, office buildings, restaurants, churches, hotel/motels, hospital/nursing homes, adult day care facility, fraternity/sorority houses, theaters, sidewalks, and parking lots/garages.

#### A.4 Perpetrator

This is categorized into spouse/partner, parent, other relative, unrelated caregiver, friend/acquaintance, official authorities, multiple perpetrators, stranger, other specified, and unknown.

#### A.5 Reason for Assault

This is categorized into altercation, robbery/burglary, drug-related, sexual assault, gang-related, other specified, and unknown.

#### A.6 Causative Agent of Injury

This is categorized into motor vehicle occupant, motorcyclist, pedal cyclist, pedestrian, other transport, fall, struck by/against, cut/pierced, overexertion, fire/burn, poisoning, inhalation/suffocation, drowning/near drowning, machinery, foreign body, dog bite, other bite/sting, firearm gunshot, BB/pellet gunshot, and natural/environmental causes.
